# How can health systems under stress achieve universal health coverage and health equity?

**DOI:** 10.1186/s12939-024-02293-2

**Published:** 2024-11-21

**Authors:** Kumanan Rasanathan

**Affiliations:** Alliance for Health Policy and Systems Research, WHO, Geneva, Switzerland

## Abstract

Health systems worldwide face an increasing array of interconnected stresses, undermining efforts to achieve Sustainable Development Goal (SDG) 3.8 on universal health coverage by 2030. The confluence of challenges—such as the COVID-19 pandemic, the climate crisis, rising conflicts, and economic pressures—has severely strained health systems, often regressing progress in service delivery and exacerbating inequities. Additionally, demographic shifts, urbanization, the rise of noncommunicable diseases, and ongoing health worker shortages add further pressure. The accompanying papers in this supplement provide case studies from various countries, illustrating the impacts of these challenges on health systems and the exacerbation of pre-existing inequities. They also highlight potential strategies for resilience, such as digitalization, reconfigured service delivery, and people-centered care. However, the resilience of health systems requires not just technological advancements but also investments in workforce, financing, supply chains, and governance – including reserve capacity in the system. Addressing health inequities is critical, as inequality undermines trust in health systems and social cohesion, which are essential for increased resourcing of health systems and resilience. As the 2030 deadline approaches, there is a need to place resilience and the ability to manage crises and stresses as part of the “everyday business” of health systems. Beyond digitalization, achieving this evolution will require social innovation, greater participation by communities in their own healthcare, prioritization of resources for underserved and marginalized population groups, and working across sectors – to enable adaptive health systems that can deliver universal health coverage even in times of stress.

Health systems are facing an increasing array of interlinked stresses and challenges that undermine equity and hinder the achievement of Sustainable Development Goal (SDG) 3.8 on universal health coverage by 2030. Many of these stresses relate to the increasingly discussed challenge of a “polycrisis.” [1] The confluence of the COVID-19 pandemic, the climate crisis, rising war and conflict, and economic and cost-of-living challenges have stretched health systems’ capacities, in some cases leading to regressions in service delivery [2]. Additionally, other challenges and transitions are placing further stress on health systems, including demographic transition with increased numbers of older people, the predominance of noncommunicable diseases due to the epidemiological transition, the location of a majority of the world’s population in cities due to urbanization, and the continuing health worker crisis.

It is now well documented that COVID-19 increased health inequities in numerous ways, affecting service delivery, health outcomes, and social determinants of health [3]. Nearly five years after the onset of the pandemic, service delivery levels for key programmes, such as immunization, have still not returned to 2019 levels [4], and the anticipated improvements that might have occurred without the pandemic have not materialized.

The impact of the climate crisis, with increasing temperatures and extreme weather, long predicted to undermine health, is becoming increasingly apparent. Health systems are struggling with heightened systemic risks for health due to climate change (including changes in vector distribution, the impact of excessive heat, and undermining of food and water security) leading to changes in burden of disease, as well as direct disruptions to health service delivery caused by heat and extreme weather events.

Recent years have seen a rise in conflict, as measured by the Uppsala Conflict Data Program (UCDP) index [5]. More of the world’s population now lives in conflict or post-conflict situations, requiring health services to be delivered in these challenging settings. Health systems are struggling to adapt to this new reality, including record levels of forced migration [6], while also increasingly facing direct attacks on health facilities during conflicts.

The economic consequences of COVID-19, coupled with the need to allocate resources towards climate change mitigation and adaptation both domestically and through multilateral finance, have significant implications for health financing. The fiscal space for increasing investment in health systems, crucial for achieving universal health coverage, is now much more constrained than it was five years ago [7]. Although overseas development assistance for health is a minor contributor to overall health spending globally, it is a crucial contribution to the weakest health systems, often in countries most affected by crises including conflict and climate change. Such assistance peaked dramatically during COVID-19 and has since reduced significantly, with projections indicating it may flatten or decrease even further through the 2030 SDG deadline [8]. 

These combined stresses have resulted in stagnation of progress towards universal health coverage, with 108 out of 194 countries either stalled or regressing on the SDG UHC targets for service coverage since 2015 [9]. This lack of progress has major implications for health equity; managing and overcoming the stresses faced by health systems is essential to achieving universal health coverage and reducing health inequities.

The papers in the accompanying supplement drawn from Latin America, Spain, Thailand, Philippines, Ghana and Nigeria illustrate how health systems are under stress. The papers provide several examples of the impacts and disruptions of COVID-19 on health systems, social determinants and health inequities – also showing how many of these inequities predated COVID-19, which exacerbated them. At the same time, the papers also highlight potential lessons and experiences through which health systems can perform better during crises and improve equity, including through reconfiguring services in crises to meet people’s needs (such as the example of COVID-19 swabbing in phone booths in Thailand) [10], use of digitalization such as telemedicine [11] and by emphasizing people-centred care. 

Health systems’ capacities to overcome these stresses and scale promising experiences from COVID-19 and other crises requires building institutionalized resilience, particularly in the ability of health systems to adapt and reconfigure themselves. The concept of health systems resilience drew attention in the aftermath of the West Africa Ebola epidemic [12], but efforts to ensure this property in the subsequent period have been weak and inconsistent – as seen by the challenges health systems faced during COVID-19. There is nothing magical about the concept of health systems resilience – it requires strength in traditional health systems dimensions such as in workforce, financing, supply chains, data systems and governance [13], which in turn requires sufficient investment in basic health systems functions.

Moreover, health systems resilience requires redundancy – spare capacity in the system that can be deployed during crises and times of stress. Such redundancy is antithetical to the conceptions of efficiency in health systems that dominate health financing and planning discourse and efforts - and challenging to mobilize when even the basic functions of health systems are underfunded and difficult to resource in many settings.

Digitalization is a promising approach to contribute to improved health systems resilience to manage current stresses and crises, and to reverse stagnation towards universal health coverage. But it is not a silver bullet or singular solution. The current moment of a critical mass in available connectivity and maturity of digital technologies, including artificial intelligence, promises to finally realize the potential of digitalization in health systems. There is increasing potential for digitalization to overcome gaps in clinical intelligence, coordination and data availability to support people-centred care, especially to enable more effective chronic care. But caution is needed to avoid exacerbating health inequities rather than improving them, and to protect against the risks of artificial intelligence. The digital divide, even as connectivity and access to technology are rapidly increasing in many settings, remains a challenge for health equity. Moreover, there remains a need for a health systems approach to digitalization whereby technology is focused on addressing systems challenges, and fragmentation and a disproportionate focus on individual applications or technologies is avoided. The question of who benefits from digitalization, includes who owns the technology and who is able to usefully access it, is a crucial consideration to manage to ensure improvements in health equity.

The equity concerns from health stresses and crises are clearly not limited to issues of digitalization, as clearly shown by the papers in this supplement. But health inequities are not just an outcome of crises and lack of health systems resilience. Health inequity and broader inequality undermine social cohesion and solidarity, trust in health systems, as well as social and economic development, and therefore the possibility of investment in health systems so that they are better equipped to manage stresses and crises. The societies and systems that were best able to protect health during the COVID-19 pandemic were those with greatest trust, cohesion and equality [14]. Without political prioritization of equity, the resources for health systems resilience will be elusive – so the evidence of the importance of health equity to prevent and manage health and societal crises must be deployed more effectively to help generate this political priority.

As the world looks towards the 2030 deadline for the SDGs, and how to address the alarming lack of progress across the agenda, including for universal health coverage, there is a need to place resilience and the ability to manage crises and stresses as part of the “everyday business” of health and social systems. While the COVID-19 pandemic may have receded as an acute stress, the climate crisis and the challenge of conflict and adverse economic circumstances will be ongoing. Health systems need to be able to deliver services and function effectively in these conditions as a normal activity. Beyond digitalization, achieving this evolution will require social innovation, greater participation by communities in their own healthcare, prioritization of resources for underserved and marginalized population groups, and working across sectors – the principles of primary health care remain relevant even as the context has changed so much since the Declaration of Alma-Ata in 1978. Health policy and systems research can play a greater role in not only documenting these challenges and promising experiences, but also informing the transformation of systems towards resilience, mobilizing the promise of digitalization and avoiding potential pitfalls, and placing equity and people-centred care as core principles for health systems that can deliver universal health coverage even in times of stress.

## Data Availability

No datasets were generated or analysed during the current study.
